# Editorial Extracts and Gleanings

**Published:** 1874-01

**Authors:** T. Curtis Smith

**Affiliations:** Ohio


### Editorial Extracts and Gleanings.

---

By T. CURTIS SMITH, M.D., OF OHIO.

*Nævus Treated by Croton Oil.*—Dr. DeSmet, communicates to the Presse Medicale Belge (November 2), an account of a case of nævus which he has successfully treated by means of croton oil. He observes that when the nævus is visible, vaccination is objectionable on account of the cicatrices which are left; and moreover, as in his own case, the child has frequently been already vaccinated. In this case the girl, five years old, had a small nævus, which was situated immediately below the lower eyelid. Numerous small superficial vessels conveyed towards it, the nævus being a little larger than a half-franc piece, and having a slightly projecting center. After perchloride of iron had been tried without avail, the croton oil was resorted to. Fifteen sewing needles were passed through a cork, so as to allow the points to project for about two millimetres, and the points were so disposed as to represent as nearly as possible the form of the nævus and the direction of its chief vessels. The cork was then dipped in croton oil, and, having been applied exactly over the nævus the points of the needles were quickly forced into the part. Painful at the moment, this inoculation only left after it a slight sense of heat and pricking. A little wadding constituted all the dressing. Next day there was some swelling a little vesication, but no pain. On two occasions, at intervals of two or three days, the part was slightly painted with croton oil. The success was complete, the nævus disappearing—so that no trace of the malady remains.—*Times and Gazette, Nov.* 15, 1873.—*Clinic, Dec.* 6, 1873.

*Ligation of the Brachial Artery—Secondary Hemorrhage on the Forty-fourth Day.*—Professor W. W. Dawson, in a clinical lecture at the Good Samaritan Hospital, Cincinnati, Ohio, presented before the class a young man at twenty-five, who, in an altercation almost seven weeks prior, had received an injury (done with a marline spike) three inches below the bend of the elbow, which entered at the radial side, passed obliquely upwards, stopping just beneath the skin one and a half inches below the internal condyle, injuring the ulnar artery—the wound being similar to that produced by a bullet. The radial artery was intact. He was first under the care of Dr. Brunning. No untoward symptom occurred from the time of injury to the morning of the forty-fourth day, when a "sudden and alarming hemorrhage" occurred, during which "he lost about two quarts of blood." This was checked by compresses, and the man sent to hospital, where he had several slighter hemorrhages, to check which the house surgeon, Dr. Steely, applied a tourniquet. The patient was brought before the class pale, feeble and weary. Respiration hurried; pulse feeble; arm around seat of injury greatly swollen and discharging "a reddish thin fluid." The occurrence of severe arterial hemorrhage "at so long a period after the receipt of the injury, is certainly very remarkable." Macloud gives one similar case.

Dr. Dawson remarked: "Three resources present themselves" for relief. "1. Amputation of the arm above the elbow. 2. Ligation of the brachial. 3. Ligation of the ulnar above and below the bleeding point." In this case "the swelling and infiltration here are great. All guides to the vessel are lost; the artery is deeply situated; to find it would require an dissection incompatible with the safety of the arm. * * * In this case I decide upon ligation of the main trunk, because, should that fail, amputation is a resource still left." This was done. "Immediately after the ligation, the hand became cold and the radial artery ceased beating. In one hour the warmth returned in the hand, the pulse eighty-five, temperature 99½." The radial pulse was established in forty-eight hours. Patient continued to improve.

[In these cases of severe secondary arterial hemorrhage, where the patient becomes fearfully exhausted from the repeated attacks, the vessel being deeply seated, the surrounding tissues enormously swollen and infiltrated, it is no easy task to dissect out the cardiac and distal ends of the bleeding vessel and ligate it securely. To fully appreciate the gravity of the situation, the surgeon has only to have such a case before him demanding his immediate attention. While it is best *ceteris paribis* to ligate at the cite of the injury, it often involves greater risk to the patient than would be incurred by going to the main trunk, as Professor Dawson did in his case. Certainly hemorrhage occurring forty-four days after receipt of injury is very rare.]

Persistence of Hymen.—Dr. Gray, in the Glasgow Medical Journal, May, 1873, reports nine carefully observed cases, proving incontestably that persistence of the hymen is compatible with the wedded state; that its destruction does not necessarily follow even the calling of a prostitute. From this it follows that the persistence of the hymen after rape is not sufficient to disprove the charge. One case was a woman married eleven years, and another twenty-four years. Both had perfect hymens. There were cases of prostitutes who had lived such a life for seven, eight and eleven years, respectively. Incidentally it is stated that none of these gave any evidence of venereal disease. In four cases of married ladies, vaginismus had prevented the rupture of the hymen.—*Detroit Review of Medicine, Nov.* 1873.

*Nitric Acid in the Treatment of Uterine Diseases.*—Dr. Atthill (London Obstetrical Journal, June, 1873) reports ten cases of uterine disease treated by strong nitric acid alone. From his experience, he concludes, (1) that when tenderness on pressure exists, it should, before the acid is applied, be removed, or at least materially lessened by local depletion; (2) that when this precaution has been taken, fuming nitric acid may be safely applied to the interior of the uterus; (3) that when the cervix has been previously freely dilated, its application does not cause any pain; (4) that in some instances it appears to have a directly soothing influence on the uterine nerves; (5) that when applied through a canula, pain is sometimes produced, but less in character than that caused by the use of the solid nitrate of silver; (6) that its use in some cases is followed by hemorrhage of moderate amount, which does not influence the result of the case; (7) that if applied to the *healthy* cervix, it may produce contraction, and perhaps obliteration of the cervical canal—hence the *healthy* cervix should be protected from its action;

(8) that in cases where imbedded fibrous tumors exist, the fuming nitric acid exercises a marked effect in controlling hemorrhage, and in allaying pain.—*Detroit Review, Nov.* 1873.

*Phosphorus Mixture.*—Dissolve a grain of phosphorus in six drachms of chloroform. Add two ounces of pure glycerin and shake well. The chloroform and glycerin form a permanent union which the addition of water does not separate. Dose, one drachm three times a day.—*Medical Archives.*

*Food for Infants.*—Dr. J. C. Nourse, in the Clinic for Dec. 18, 1873, says, "A lady in my practice having lost three children within six months of their births from chronic diarrhœa as a consequence of artificial lactation, I urged her to try the food recommended by Dr. Jacobi, of New York, in the case of an infant three months old, who was having from twelve to fifteen discharges every twenty-four hours as the result of artificial feeding, and to all appearances would die. I say urged, for the mother had a dread of diluted milk in any form, and in this case, but without success, was using cracker water, diluted cream, etc. She gave the food as directed, and now, after months' use, we have a hearty growing babe, without diarrhœa—all as a result of the food given. I have recommended it in several other cases, and am confident it is the best artificial food that can be used. It is prepared as follows: Crack a teaspoonful of baıley in a common coffee-mill, then boil it fifteen minutes in a gill of water, adding a pinch of salt. Then strain, and for a young child add half as much cow's milk as you have barley-water, and give while tepid from a nursing bottle. Sweeten lightly with sugar. If the bowels are costive, oat-meal should be used instead of barley. *Keep the bottle clean*" (italics ours.)

*Encysted Tumor of the Neck.*—Dr. C. W. McMillen, (Clinic December 18, 1873), reports a case of double encysted tumor of the neck in a child, so large that it crowded the head against the opposite shoulder. After preparatory treatment he punctured the anterior half of the tumor with a grooved needle and bistoury, from whch clear serum issued. He then punctured the posterior portion, which was more dense, in the same manner. From this there issued "a thick grumous coffee-ground fluid." This gave great relieve for two or three months, but the sacks refilled. He then passed a double tread of silk so as to enter both cysts. The fluid issued slowly from the openings thus made. After a time suppuration ensued, when the bistoury was again used evacuating a large quantity "of offensive pus. The wall collapsed," and recovery followed.

*Frequently Recurring Epistaxis Relieved by Ergot after Local Treatment had Failed.*—Dr. Andrew H. Smith, of New York city, (Record, October 15, 1873), reports a case of this kind. The patient was naturally of rather a delicate organization. Rhimoscopic examination only showed the mucus membrane of the left side "unduly red." Astringents in variety were used locally, and such general treatment used as symptoms demanded. This improved the general health, and was continued for two weeks. The nasal congestion had disappeared. "Still the hemorrhages recurred as frequently as ever." Fluid extract ergot in gtts xx. ter die. was then given for ten days, which checked the trouble. The agent was then stopped and the bleeding recurred, but a second resort to it, made a permanent check to the trouble. The ergot was gradually diminished and its use stopped entirely after a month.

